# Cancer incidence in the Ecuadorian Amazon: A retrospective study (1990–2019)

**DOI:** 10.1371/journal.pone.0359707

**Published:** 2026-10-05

**Authors:** Miguel San Sebastián, Osvaldo Fonseca-Rodriguez, Wilmer Tarupi, Sara Castel-Feced

**Affiliations:** 1 Department of Epidemiology and Global Health, Umeå University, Umeå, Sweden; 2 Instituto de Epidemiología y Salud Comunitaria “Manuel Amunárriz”, Hospital Franklin Tello, Nuevo Rocafuerte, Orellana, Ecuador; 3 Quito Cancer Registry, Sociedad de Lucha contra el Cáncer, SOLCA, Quito, Ecuador; 4 Department of Statistical Methods, Zaragoza University, Zaragoza, Spain; Universidade dos Açores Departamento de Biologia: Universidade dos Acores Departamento de Biologia, PORTUGAL

## Abstract

**Background:**

There is a scarcity of cancer studies in the Amazon region. This study aimed to analyse trends in cancer incidence within the Ecuadorian Amazon between 1990 and 2019, identifying the most prevalent cancer types and assessing their provincial and temporal distribution.

**Methods:**

A retrospective analysis was conducted using data from the Quito population-based cancer registry, focusing on patients whose habitual residence was in the Amazonian provinces of Sucumbíos, Orellana, Napo, and Pastaza. Age- and sex-standardised incidence rates were calculated, employing Segi’s world standard population as the reference.

**Results:**

A total of 4,881 cancer cases were identified, revealing a 357% increase in the number of recorded cancer cases from the first to the last study period. Among men, the most common cancer types were stomach, prostate, and non-melanoma skin cancers, whereas in women, cervical, breast, and thyroid cancers predominated. Notably, there was a particularly high increase in prostate and breast cancer cases.

**Conclusions:**

The findings indicate an important increase in recorded cancer cases and age-standardized incidence rates in the Ecuadorian Amazon, highlighting the urgent need to enhance healthcare infrastructure and cancer registry systems in the region. Targeted prevention and early detection programmes tailored to the specific needs of this population are urgently needed.

## Introduction

Cancer remains a leading cause of mortality globally, accounting for nearly 10 million deaths in 2022. The cancers with the highest incidence worldwide include breast, lung, colorectal, and prostate cancers [1]. Effective prevention and control of these diseases necessitate robust information systems. Population-based cancer registries play a pivotal role as continuous surveillance mechanisms, systematically collecting data on new cancer cases within defined populations. These registries are essential for calculating incidence and mortality rates and serve as reliable sources for health policy planning and impact assessment [2].

The Amazon region, one of the most biodiverse and expansive areas globally, spans approximately 7.5 million square kilometers across nine countries and harbors about 10% of all known species. It is home to numerous Indigenous communities and rural populations with historical migration patterns. The Amazon basin has a very low population density overall, with fewer than six people per square kilometre on average (around 3–4 in Brazil’s part). This is due to challenging rainforest conditions, difficult terrain and poor agricultural soil, with most people concentrated in urban centres [3]. However, the Amazon faces multiple challenges, including deforestation, illegal mining, wildlife trafficking, and climate change, which threaten both its biodiversity and the well-being of its inhabitants. In this complex context, cancer in the Amazon region represents a multifaceted challenge, intertwining environmental, socioeconomic, healthcare access, and case registration factors. Understanding these interactions is crucial for effectively addressing this public health issue [4,5].

Evidence on cancer in the Amazon region remains scarce. A recent study among Indigenous populations in the Brazilian Amazon found that cervical, stomach, and liver cancers accounted for 52% of cases between 2000 and 2012, with higher incidence rates compared to non-Indigenous counterparts. The study highlighted that socioeconomic vulnerability and limited access to healthcare services among Indigenous populations could increase the risk of infection-related cancers, such as HPV for cervical cancer, *H. pylori* for stomach cancer, and viral hepatitis for liver cancer [6]. Another study in the same region (Acre state) indicated that, compared to non-Indigenous populations, mortality rates for liver, stomach, colorectal cancers, and leukemias were more than double among Indigenous men; among Indigenous women, cervical, stomach, liver cancers, and leukemias were 30% higher [7]. Additionally, a study from the same Brazilian state showed a significant increase in breast cancer mortality rates but relative stability in cervical cancer rates from 1980 to 2014, highlighting concerns regarding healthcare access and follow-up [8].

Information on cancer in the Ecuadorian Amazon is equally scarce. A report covering 1985–1998 indicated lower cancer incidence rates in the Amazon region compared to Quito’s population. The age-standardized incidence rate was 39.49 per 100,000 men and 68.25 per 100,000 women. The most common cancers among men were stomach, skin, lymph nodes, hematopoietic system, and prostate; among women, cervical, stomach, breast, skin, and hematopoietic system cancers were predominant [9]. A subsequent update covering 1985–2003 observed an increase in cancer incidence during the first decade (1985–1994), followed by a decrease in the second (1995–2003). During the last period (2000–2003), the highest age-standardized annual incidence rates among women were for cervical (19.9/100,000) and stomach (5.9/100,000) cancers; among men, stomach cancer was most frequent, with 7.7 cases per 100,000 inhabitants [10]. An ethnic-comparative study conducted in 2002 revealed similar or even lower risks for various cancer types among Indigenous populations compared to non-Indigenous populations in the same Amazon region. Indigenous men exhibited lower risks for stomach, skin, prostate, lymph node cancers, and leukemia; similarly, Indigenous women had lower risks for stomach, skin, breast, cervical, and lymph node cancers [11]. These findings must be interpreted considering the limited access of indigenous populations to healthcare services and, consequently, to cancer registration.

The limited existing literature underscores the urgency of updating cancer incidence data in Ecuador’s Amazon region. In this context, the present study aims to analyse trends in cancer incidence between 1990 and 2019, identifying the most prevalent cancer types and assessing their provincial hic and temporal distribution

## Methods

### Study design

This study was designed as a retrospective, registry-based ecological analysis using data from the Quito Population-Based Cancer Registry. The unit of analysis was aggregated population-level cancer incidence data stratified by province, sex, age group, and five-year study period. The study population included residents of the Ecuadorian Amazon provinces of Sucumbíos, Orellana, Napo, and Pastaza diagnosed with cancer between 1990 and 2019 and registered in the NCR.

### Study population

This study utilised data from patients diagnosed with cancer in Quito, whose habitual residences were in the Ecuadorian Amazon provinces of Sucumbíos, Orellana, Napo, and Pastaza. According to estimates from the National Institute of Statistics and Censuses (INEC in Spanish) for the year 2023, these provinces have a combined population of approximately 627,195 individuals, with women constituting 48.1%. Both Ecuador as a whole and these four provinces have experienced significant demographic shifts. Census data indicate a narrowing of the population pyramid base and an expansion of the older age groups. In 1990, 66.4% of the population in these provinces was under 25 years of age; by 2019, this proportion had declined to 54.4%. Conversely, the proportion of individuals aged 65 years or older increased from 2.3% in 1990 to 4.6% in 2019 [12].

The provinces of Morona Santiago and Zamora Chinchipe were excluded from the study due to their populations’ tendency to seek medical care in the Southern Highlands and Coastal regions, resulting in a low number of cancer cases recorded in the Quito NCR from these areas (Fig 1).

**Fig 1:**
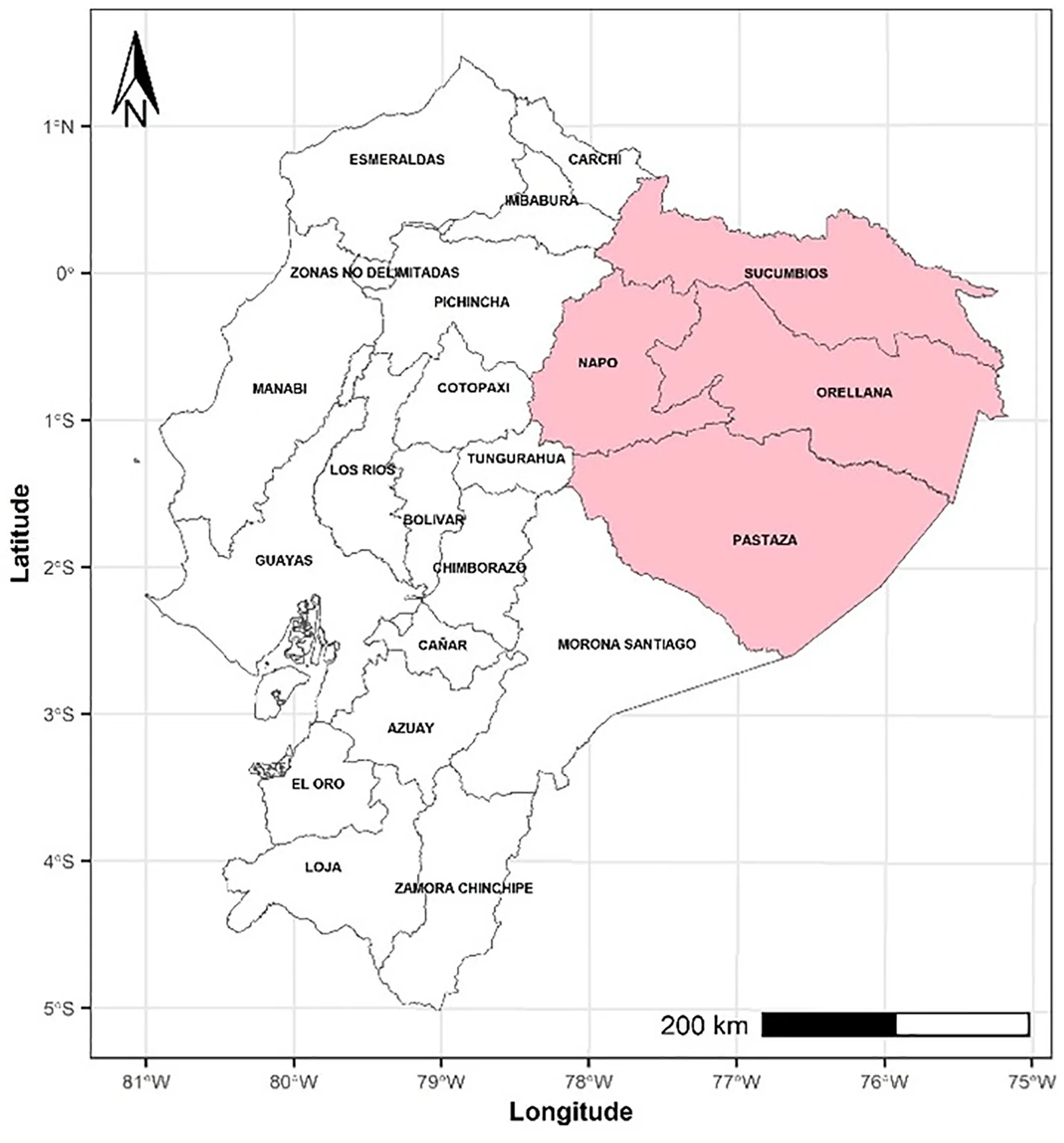
Map of the study area; in pink, the four provinces of the study (created by the authors using geographic data from the Ecuadorian National Institute of Statistics and Censuses, available under a Creative Commons Attribution 4.0 International Licence).

Although each of the four provinces has a provincial-level hospital, none offers histopathological services or access to oncological treatment. In addition, no organized cancer prevention programmes are currently available in the region.

### Cancer data

The Amazon region lacks its own cancer registries. Consequently, case ascertainment in the region depends heavily on referral pathways, access to specialized diagnostic services, and the capacity of patients to reach healthcare facilities in Quito, the nation’s capital. Geographic, socioeconomic, and healthcare access barriers may therefore contribute to incomplete case capture and differential underreporting across provinces and over time. All cases diagnosed in Quito are recorded in the Quito Population-Based Cancer Registry, which served as the data source for this study.

The Quito Population-Based Cancer Registry collects information on all new cancer cases among patients whose habitual residence is in Quito. It also processes data on all cases diagnosed in Quito, regardless of the patient’s place of residence. The registry’s information sources include histopathological reports from pathology and haematology laboratories, as well as medical records from oncology care centres within the Public Integrated Health Network and the Complementary Network.

Additionally, the Quito Population-Based Cancer Registry has access to hospital discharge and mortality data collected by INEC, facilitating the identification of new cancer cases. The registry is a member of the International Association of Cancer Registries (IACR) and adheres to the standards established by the International Agency for Research on Cancer (IARC), which set global criteria for assessing data quality and reliability [13].

Each individual record in the Quito Population-Based Cancer Registry includes a personal identification code, sex, age at diagnosis, cancer site, histology according to the International Classification of Diseases, 10th Revision (ICD-10), year of diagnosis, place of residence at diagnosis, and the patient’s educational level. This study included all cases recorded in the registry corresponding to the provinces of Sucumbíos, Orellana, Napo, and Pastaza during the period 1990–2019. Data were accessed on 1 October 2023.

Based on the preceding information, we designed a population-based registry ecological study, with analyses stratified by province and time period.

### Ethics statement

This study used anonymized data from the Quito Population-Based Cancer Registry database and adhered to the principles of good clinical practice (GCP) and the Declaration of Helsinki. Since participant identification was not possible but personal information was accessed, ethics committee approval was sought and granted by the SOLCA Quito Research Ethical Committee (reference OBS.23.216).

### Analysis

Initially, the total number of cancer cases were calculated by sex, province, and study period. To ensure sufficient case numbers in each group, the analysis was structured into six five-year periods: 1990–1994, 1995–1999, 2000–2004, 2005–2009, 2010–2014, and 2015–2019. Subsequently, incidence rates for all cancers combined were estimated, stratified by sex. For the denominator in rate calculations, population data for the four provinces were used, stratified by sex and five-year age groups for each period. The estimated mid-period population, multiplied by five, served as the denominator. These data were obtained from INEC projections based on the national censuses of 1990, 2000, and 2010.

Age-standardised incidence rates for all cancers combined were then calculated by province and period, using the direct standardisation method and Segi’s world standard population as the reference [14]. To better describe the current cancer burden, age-standardised incidence rates for the most recent period (2015–2019) were also calculated by province, for all cancers combined and the five most common cancer types, stratified by sex.

Temporal trends in overall cancer incidence were assessed using quasi-Poisson regression models to account for overdispersion in the count data. The number of incident cancer cases was specified as the dependent variable, with the logarithm of the population included as an offset term. Calendar period was entered as an ordinal variable representing consecutive 5-year intervals, and models were adjusted for age group and sex. Incidence rate ratios (IRRs), 95% confidence intervals (CIs), and p-values for trend were estimated to quantify changes in incidence over time. All analyses were conducted using the open-source statistical software R [15].

## Results

### Cancer incidence

Between 1990 and 2019, a total of 5,008 cancer cases were recorded across the four Amazonian provinces of Sucumbíos, Orellana, Napo, and Pastaza. However, due to missing values in some variables, the analysis was based on 4,881 cases, 36.8% of which were men. The 127 missing cases were due to lack of information on age (n = 13) and the name of the province (n = 114).

The data reveal a progressive increase in the number of recorded cases over successive five-year periods, culminating in a 357% rise between the first (1990–1994) and the last (2015–2019) study periods. While both sexes exhibited similar percentage increases, men experienced more pronounced rises during 1995–1999 and 2005–2009, whereas women showed greater increases in 2005–2009 and 2010–2014 (Table 1).

**Table 1:** Total number of recorded cancer cases and percentage increase (in parentheses, relative to the preceding period) by period and sex.

	1990-1994	1995-1999	2000-2004	2005-2009	2010-2014	2015-2019
Total	328	437 (33.2)	534 (22.2)	852 (59.6)	1 231 (44.5)	1 499 (21.8)
Men	124	177 (42.7)	215 (21.5)	314 (46.0)	434 (38.2)	532 (22.6)
Women	204	260 (27.5)	319 (22.7)	538 (68.7)	797 (48.1)	967 (21.3)

The four most common cancer types by sex are detailed in Tables 2 and 3. These cancers accounted for approximately 40% of all cases in men and 60% in women throughout the study periods. In men, the most frequent cancers were prostate, stomach, skin (non-melanoma), and lymphoid leukemia. A declining trend in stomach cancer incidence was observed, while prostate cancer showed a progressive increase over time. Among women, the predominant cancers were invasive cervical, breast, thyroid, cervical in situ, and skin (non-melanoma). A decrease in cervical cancer incidence was noted, whereas breast and thyroid cancers exhibited sustained increases.

**Table 2:** Number of cases and proportion (%) of the four most frequent cancers in men by period (ICD-10 codes in parentheses).

	1990-1994	1995-1999	2000-2004	2005-2009	2010-2014	2015-2019
Stomach (C16)	27 (21.8)	29 (16.4)	39 (18.1)	47 (15.0)	43 (9.9)	52 (9.8)
Non-melanoma skin (C44)	15 (12.1)	16 (9.0)	24 (11.2)	35 (11.2)	45 (10.4)	44 (8.3)
Prostate (C61)	–	17 (9.6)	16 (7.4)	40 (12.7)	59 (13.6)	73 (13.7)
Lymphoid leukemia (C91)	7 (5.7)	–	17 (7.9)	16 (5.1)	26 (6.0)	40 (7.5)
Others	75 (60.4)	115 (65.0)	119 (55.4)	176 (56.0)	261 (60.1)	323 (60.7)

**Table 3:** Numbers of cases and proportion (%) of the four most frequent cancers in women by period (ICD-10 codes in parentheses).

	1990-1994	1995-1999	2000-2004	2005-2009	2010-2014	2015-2019
Invasive cervix uteri (C53)	59 (28.9)	69 (26.5)	87 (27.3)	111 20.6	121 (15.2)	177 (18.3)
Thyroid (C73)	–	–	–	26 4.8	74 (9.3)	131 (13.6)
Breast (C50)	16 (7.8)	20 (7.7)	32 (10.0)	53 10.0	91 (11.4)	130 (13.4)
Cervix uteri in situ (D06)	23 (11.3	37 (14.2)	22 (6.9)	135 25.1	146 (18.3)	94 (9.7)
Non-melanoma skin (C44)	15 (7.4)	–	24 (7.5)	29 5.4	51 (6.4)	64 (6.6)
Others	91 (44.6)	134 (51.5)	154 (48.3)	184 34.2	314 (39.4)	371 (38.4)

### Age-standardised incidence rates

Fig 2 illustrates the age-standardised incidence rates per 100,000 inhabitants for the entire Amazon region and each province across the study periods. Overall, there was a progressive increase in age-standardized cancer incidence rates for both sexes, with a more pronounced rise in the last three periods. Among men, the incidence rate escalated from 19 per 100,000 in 1990–1994–50 per 100,000 in 2015–2019, marking a 163.2% increase. For women, the rate rose from 37 to 96 per 100,000, reflecting a 159.5% increase. Detailed crude and age-standardised rates for the entire region and by province are available in S1 Table in S1 Appendix.

**Fig 2:**
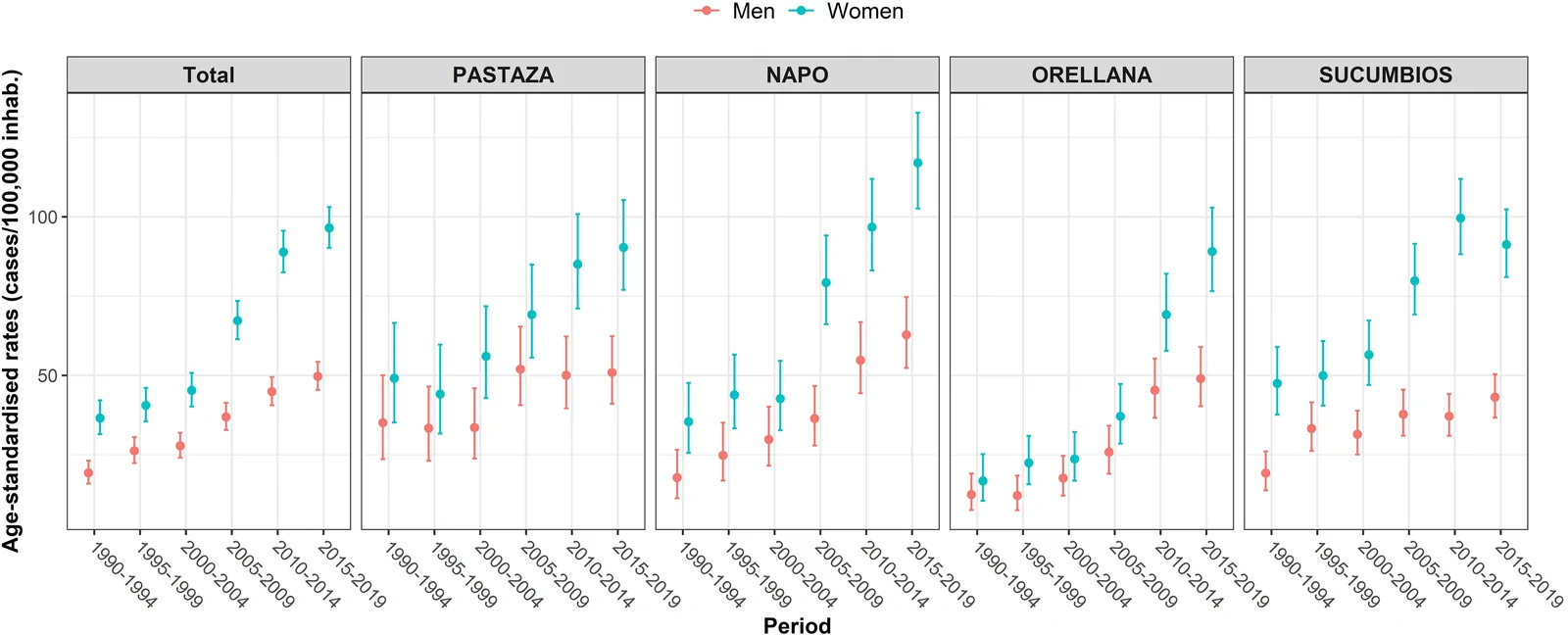
Annual average age-standardised incidence rates (adjusted to the world population) for the total Amazon region (four provinces) and each province.

While a general upward trend was observed across all provinces, variations were noted. In women, the provinces of Pastaza, Napo, and Sucumbíos experienced more significant increases in the last three periods. Similarly, in men, Napo and Orellana showed more pronounced rises in cancer incidence rates during recent periods.

### Most frequent cancers in 2015–2019

Table 4 presents the crude and age-standardised incidence rates per 100,000 inhabitants, along with 95% confidence intervals (CI), for the five most frequent cancers by sex during the 2015–2019 period. In men, prostate cancer was the most prevalent (8.3 per 100,000), followed by stomach cancer (5.4 per 100,000) and non-melanoma skin cancer (4.9 per 100,000). Among women, invasive cervical cancer was most common (18.2 per 100,000), followed by breast cancer (13.6 per 100,000) and thyroid cancer (11.8 per 100,000).

**Table 4:** Annual average crude and age-standardised incidence rates (per 100,000 inhabitants) with 95% confidence intervals for the five most frequent cancers by sex in the Amazon region during 2015–2019.

	MEN		WOMEN	
Cancer type	Crude rate	Age-standardised rate (x 100 000)	Crude rate	Age-standardised rate (x 100 000)
Stomach (C16)	3.3	5.4 (3.98–7.07)	–	–
Non-melanoma skin (C44)	2.8	4.9 (3.52–6.54)	4.4	7.9 (6.06–10.15)
Breast (C50)	–	–	9.0	13.6 (11.30–16.24)
Cervix uteri in situ (D06)	–	–	6.5	7.8 (6.22–9.62)
Invasive Cervix uteri (C53)			12.2	18.2 (15.48–21.17)
Prostate (C61)	4.7	8.3 (6.47–10.40)	–	–
Thyroid (C73)	–	–	9.1	11.8 (9.75–14.08)
Non-follicular linfoma (C83)	1.9	2.8 (1.84–3.98)	–	–
Lymphoid leukemia (C91)	2.6	2.5 (1.74–3.41)		

### Trend test in overall cancer incidence

In age- and sex-adjusted quasi-Poisson regression models, cancer incidence increased significantly over time, with an estimated 22.8% increase per 5-year period (IRR = 1.23, 95% CI 1.20–1.25). Overall, incidence was higher in women than in men (IRR = 2.00, 95% CI 1.88–2.14). However, a significant interaction between sex and period was observed (interaction IRR = 1.04, p = 0.050), indicating that incidence increased more rapidly in women than in men over the study period. Specifically, incidence rose by approximately 19.7% per 5-year period in men and 24.7% per 5-year period in women. Detailed estimates and their 95% CI are provided in S2 Table in S2 Appendix.

## Discussion

This study provides an updated analysis of cancer incidence in the northern Amazon region of Ecuador from 1990 to 2019, utilizing data from the Quito Population-Based Cancer Registry. The findings indicate a significant increase in cancer incidence across the region, with notable rises in prostate, stomach, and non-melanoma skin cancers among men, and invasive cervical (malignant neoplasm of the cervix uteri), breast, and thyroid cancers among women.

The observed upward trend, particularly pronounced in the last three five-year periods (2005–2019), underscores the urgent need for a comprehensive cancer control program in the region. The increase in cancer cases may reflect a combination of factors, including actual growth in disease prevalence, demographic shifts towards older age groups, improved cancer registration practices, and enhanced access to healthcare services.

The scarcity of studies in the Amazon region and methodological differences in data collection make it difficult to compare with other countries in the region. Comparative analysis with other Latin American countries reveals similarities in prevalent cancer types, such as prostate, lung, stomach, colorectal, and bladder cancers in men, and breast, cervical, colorectal, stomach, and lung cancers in women. However, studies focusing on Indigenous populations suggest a higher prevalence of cervical, stomach, prostate, and gallbladder cancers [5].

In the case of the Amazon region, most available studies originate from Brazil, where research has specifically focused on Indigenous populations. However, in Ecuador, conducting a similar analysis is currently unfeasible, as the Quito Population-Based Cancer Registry does not collect information on ethnic identity. For illustrative purposes, a study conducted among Indigenous populations in the state of Acre, located in the Brazilian Amazon, revealed that the most frequent cancers among men were stomach, liver, colorectal, leukemia, and prostate cancers. Among women, the most prevalent were cervical, stomach, leukemia, liver, and breast cancers [6]. This indicates a cancer profile that is partially different from that observed in our region.

The most common cancer types in Ecuador’s Amazon region mirror those observed at the national level. However, incidence rates in the Amazon are lower than those reported in Quito [16]. While this disparity might suggest a reduced cancer risk in the Amazon, it is more plausibly attributed to significant underreporting. This underreporting likely results from limited diagnostic infrastructure and challenges in accessing specialized medical services in the region.

Of particular concern are the marked increases in prostate cancer among men, and breast and thyroid cancers among women, as well as the relative stability in the incidence of other tumors, especially given the feasibility of implementing prevention programs for most of these cancer types.

The marked increase in prostate cancer incidence among men should also be interpreted with caution. In addition to population ageing, increases in prostate cancer incidence observed internationally have frequently been linked to greater use of prostate-specific antigen (PSA) testing, improved diagnostic access, and changes in health-seeking behavior [17]. Therefore, part of the increase observed in the Amazon region may reflect improved detection and diagnostic activity rather than only changes in underlying cancer risk. Notably, despite the upward trend, prostate cancer incidence in the Amazon remained considerably lower than that reported in Quito, where incidence rates are approximately 63 cases per 100,000 population and have remained relatively stable over time [18]. This difference may partly reflect variations in healthcare access, socioeconomic conditions and lifestyle-related risk factors between the two regions.

Breast cancer incidence also increased progressively during the study period. This pattern is consistent with trends observed in Latin American countries undergoing demographic, social, and epidemiological transitions. Several factors may contribute to this increase, including population ageing, changes in reproductive patterns, urbanization, lifestyle changes related to diet, obesity, and physical inactivity, as well as improved access to diagnostic services and greater awareness leading to increased detection [19,20]. In the Amazon region, improvements in referral pathways and healthcare access over time may also have contributed to the increasing number of diagnosed cases. A notable finding was the increasing incidence of thyroid cancer among women in the Amazon, where it emerged as the the third most common cancer type. This pattern aligns with national trends in Ecuador, where thyroid cancer is the second most frequent diagnosed cancer among women, after breast cancer. Ecuador has one of the highest thyroid cancer incidence rates in Latin America, and has been reported to have the highest incidence among women in the region [21,22]. Internationally, the rising incidence of thyroid cancer has often been associated with greater use of diagnostic imaging, incidental detection of small tumours, diagnostic expansion, and possible overdiagnosis rather than solely changes in underlying disease occurrence [23,24]. Although these mechanisms cannot be directly evaluated in the present study, improvements in access to diagnostic services and imaging technologies over time may have contributed to the observed increase. Nonetheless, thyroid cancer has received limited attention in studies conducted in the Amazon region or among Indigenous populations [5,6]. Further research is therefore needed to better understand the epidemiology and determinants of thyroid cancer in the region.

The coexistence of relatively high rates of both invasive cervical cancer and cervical carcinoma in situ may reflect complex and overlapping processes, including changes in screening coverage, diagnostic practices, and access to gynecological services. Since cervical cancer is strongly associated with persistent HPV infection and socioeconomic inequalities, the observed patterns may also indicate uneven access to preventive interventions, including HPV vaccination, Pap smear screening, and timely treatment of precancerous lesions across the region [25].

It is also essential to further examine social inequalities in cancer within this region, considering the substantial evidence highlighting socioeconomic, ethnic, and geographic disparities in cancer incidence and mortality [26]. These disparities represent an additional challenge for the state and its institutional healthcare response capacity, particularly in ensuring that health interventions are distributed equitably. In the Ecuadorian context, this situation is further complicated by the strong segmentation and fragmentation of the healthcare system, factors that, unfortunately, tend to exacerbate existing social health inequities.

### Methodological considerations

Several limitations should be considered when interpreting the study’s findings. Population estimates were based on projections from national censuses conducted in 1990, 2000, and 2010. Although these are the best available data, they may not fully capture demographic changes that occurred during the study period, particularly in a region characterized by migration, population growth, and demographic transition. Consequently, some uncertainty may exist in the denominator estimates used for incidence calculations.

The Ecuadorian Amazon does not have its own population-based cancer registry. Cancer cases are therefore captured through the Quito Population-Based Cancer Registry only when patients are referred, diagnosed, and registered within the healthcare system linked to Quito.

Although the Quito Population-Based Cancer Registry has a high completeness rate (95%), substantial geographical, socioeconomic and infrastructure barriers in the Amazon region, particularly in remote and Indigenous communities, may contribute to underreporting and incomplete case ascertainment. Furthermore, referral systems, diagnostic infrastructure, pathology services, transportation access, and healthcare utilization likely changed considerably between 1990 and 2019. Consequently, part of the observed increase in recorded cancer cases and incidence rates may reflect improvements in diagnostic access, referral capacity, and registry completeness over time rather than only changes in underlying cancer occurrence. These factors may also vary between provinces, potentially contributing to differential underreporting and ascertainment bias across the study region.

The study employed an ecological design based on aggregated population-level data. Therefore, the findings should be interpreted as reflecting population-level patterns rather than individual-level risks, and the possibility of ecological fallacy cannot be excluded. In addition, the use of secondary data constrained the ability to control for potential confounders. Information on individual-level risk factors and socioeconomic characteristics was not consistently available, which may have resulted in residual confounding.

Several sources of information bias should also be considered. Inaccuracies in reported residence information may have resulted in geographic misclassification of some cases. In addition, the absence of ethnic identifiers in the registry prevented analyses by ethnic group, limiting the ability to examine potential disparities between Indigenous and non-Indigenous populations. Finally, because data were compiled from multiple sources, some variability in data completeness and quality may have occurred over time and across cantons. Although all data sources followed standardized protocols and definitions, some degree of information bias cannot be ruled out.

## Conclusions

The study documents a substantial increase in both recorded cancer cases and age-standardized incidence rates in Ecuador’s Amazon region from 1990 to 2019. The most common cancers among men were prostate, stomach, and non-melanoma skin cancers while cervical, breast, and thyroid cancers predominated among women. The significant rise in prostate and breast cancer cases is particularly concerning.

Although these findings should be interpreted in light of possible changes in healthcare access, diagnostic capacity, and registry completeness over time, they highlight the need to strengthen cancer surveillance, improve access to diagnostic and treatment services, and support cancer prevention and early detection efforts in the region. Such initiatives should be integrated within a national health system framework that ensures universality, quality, and equity in healthcare services.

## Supporting information

S1 Appendix Table S1. Crude and standardised rates with confidence intervals (CI) (annual incidence) for all cancers in total, by province and sex in each of the periods studied. (DOCX)

S2 Appendix Table S2. Results of the quasi-Poisson regression analysis of overall cancer incidence by calendar period, age group, and sex, Ecuador, 1990–2019. (DOCX)
