# Supplementary material for: Cancer incidence in the Ecuadorian Amazon: A retrospective study (1990–2019)
Source: PLoS One. 2026 Oct 5;21(10):e0359707. doi: 10.1371/journal.pone.0359707 (PMC13637904; doi:10.1371/journal.pone.0359707)
Supplement: Supplementary file 1 [file pone.0359707.s001.docx]

***APPENDIX 1***

***Table S1. Crude and standardised rates with confidence intervals (CI) (annual incidence) for all cancers in total, by province and sex in each of the periods studied.***

| PROVINCE | PERIOD | CRUDE RATE | STANDARDISED RATE (95% CI) |
| --- | --- | --- | --- |
| **MEN** |  |  |  |
| Total | 1990-1994 | 13.91 | 19.3 (15.9-23.15) |
| Total | 1995-1999 | 17.83 | 26.3 (22.38-30.58) |
| Total | 2000-2004 | 19.39 | 27.8 (24.08-31.95) |
| Total | 2005-2009 | 25.28 | 36.9 (32.79-41.38) |
| Total | 2010-2014 | 31.05 | 44.9 (40.61-49.51) |
| Total | 2015-2019 | 34.04 | 49.7 (45.43-54.3) |
| Napo | 1990-1994 | 13.90 | 17.8 (11.3-26.57) |
| Napo | 1995-1999 | 16.37 | 24.8 (16.87-35.08) |
| Napo | 2000-2004 | 20.34 | 29.8 (21.56-40.15) |
| Napo | 2005-2009 | 26.39 | 36.4 (27.89-46.67) |
| Napo | 2010-2014 | 36.09 | 54.8 (44.4-66.82) |
| Napo | 2015-2019 | 42.89 | 62.8 (52.33-74.69) |
| Orellana | 1990-1994 | 8.43 | 12.5 (7.61-19.09) |
| Orellana | 1995-1999 | 8.17 | 12.2 (7.54-18.42) |
| Orellana | 2000-2004 | 11.96 | 17.6 (12.14-24.67) |
| Orellana | 2005-2009 | 15.62 | 25.8 (19.02-34.14) |
| Orellana | 2010-2014 | 28.74 | 45.3 (36.67-55.32) |
| Orellana | 2015-2019 | 30.66 | 49 (40.3-58.98) |
| Pastaza | 1990-1994 | 24.52 | 35.1 (23.63-50.04) |
| Pastaza | 1995-1999 | 24.62 | 33.4 (23.1-46.5) |
| Pastaza | 2000-2004 | 23.09 | 33.6 (23.78-45.97) |
| Pastaza | 2005-2009 | 38.54 | 52 (40.65-65.39) |
| Pastaza | 2010-2014 | 36.82 | 50.1 (39.64-62.29) |
| Pastaza | 2015-2019 | 37.28 | 50.9 (41.05-62.36) |
| Sucumbíos | 1990-1994 | 14.28 | 19.2 (13.77-26.06) |
| Sucumbíos | 1995-1999 | 23.92 | 33.3 (26.24-41.53) |
| Sucumbíos | 2000-2004 | 23.24 | 31.4 (25.07-38.9) |
| Sucumbíos | 2005-2009 | 26.21 | 37.8 (31-45.53) |
| Sucumbíos | 2010-2014 | 27.28 | 37.1 (30.95-44.18) |
| Sucumbíos | 2015-2019 | 29.99 | 43.2 (36.7-50.39) |
|  |  |  |  |
| **WOMEN** |  |  |  |
| Total | 1990-1994 | 24.84 | 36.6 (31.48-42.18) |
| Total | 1995-1999 | 28.39 | 40.6 (35.52-46.09) |
| Total | 2000-2004 | 31.15 | 45.3 (40.2-50.8) |
| Total | 2005-2009 | 46.75 | 67.3 (61.42-73.49) |
| Total | 2010-2014 | 61.63 | 88.9 (82.51-95.59) |
| Total | 2015-2019 | 66.82 | 96.5 (90.19-103.08) |
| Napo | 1990-1994 | 26.51 | 35.4 (25.59-47.63) |
| Napo | 1995-1999 | 31.87 | 43.9 (33.35-56.58) |
| Napo | 2000-2004 | 30.70 | 42.7 (32.72-54.6) |
| Napo | 2005-2009 | 56.74 | 79.3 (66.15-94.13) |
| Napo | 2010-2014 | 70.09 | 96.7 (83.09-111.94) |
| Napo | 2015-2019 | 82.73 | 116.4 (102-132.11) |
| Orellana | 1990-1994 | 10.31 | 16.8 (10.51-25.19) |
| Orellana | 1995-1999 | 16.09 | 22.4 (15.69-30.94) |
| Orellana | 2000-2004 | 16.27 | 23.7 (16.83-32.17) |
| Orellana | 2005-2009 | 23.52 | 37.1 (28.52-47.31) |
| Orellana | 2010-2014 | 44.39 | 69.2 (57.75-82.07) |
| Orellana | 2015-2019 | 57.59 | 89.1 (76.57-102.91) |
| Pastaza | 1990-1994 | 34.23 | 49.1 (35.17-66.56) |
| Pastaza | 1995-1999 | 29.71 | 44.1 (31.68-59.71) |
| Pastaza | 2000-2004 | 38.66 | 56 (42.85-71.82) |
| Pastaza | 2005-2009 | 48.74 | 69.2 (55.62-84.95) |
| Pastaza | 2010-2014 | 61.49 | 85.1 (71.11-100.87) |
| Pastaza | 2015-2019 | 66.81 | 91.1 (77.64-106.11) |
| Sucumbíos | 1990-1994 | 32.13 | 47.5 (37.65-59) |
| Sucumbíos | 1995-1999 | 35.88 | 50 (40.48-60.85) |
| Sucumbíos | 2000-2004 | 39.73 | 56.5 (46.99-67.32) |
| Sucumbíos | 2005-2009 | 57.70 | 79.8 (69.23-91.5) |
| Sucumbíos | 2010-2014 | 69.42 | 99.6 (88.21-111.94) |
| Sucumbíos | 2015-2019 | 63.84 | 91.2 (81-102.36) |
|  |  |  |  |
| **TOTAL** |  |  |  |
| Total | 1990-1994 | 19.15 | 27.1 (24.1-30.31) |
| Total | 1995-1999 | 22.90 | 32.8 (29.65-36.17) |
| Total | 2000-2004 | 25.04 | 35.8 (32.65-39.06) |
| Total | 2005-2009 | 35.61 | 50.7 (47.22-54.4) |
| Total | 2010-2014 | 45.75 | 65 (61.26-68.9) |
| Total | 2015-2019 | 49.80 | 71.1 (67.34-74.91) |
| Napo | 1990-1994 | 20.09 | 26.2 (20.26-33.28) |
| Napo | 1995-1999 | 23.98 | 34.1 (27.41-41.91) |
| Napo | 2000-2004 | 25.43 | 36.1 (29.51-43.64) |
| Napo | 2005-2009 | 41.29 | 56.8 (49.06-65.48) |
| Napo | 2010-2014 | 52.79 | 75.2 (66.57-84.6) |
| Napo | 2015-2019 | 62.45 | 88.5 (79.65-98.07) |
| Orellana | 1990-1994 | 9.31 | 14.3 (10.34-19.28) |
| Orellana | 1995-1999 | 11.90 | 16.8 (12.7-21.63) |
| Orellana | 2000-2004 | 13.99 | 20.3 (15.88-25.46) |
| Orellana | 2005-2009 | 19.36 | 30.6 (25.21-36.85) |
| Orellana | 2010-2014 | 36.12 | 55.6 (48.59-63.37) |
| Orellana | 2015-2019 | 43.36 | 66.4 (58.97-74.38) |
| Pastaza | 1990-1994 | 29.34 | 42 (32.76-52.91) |
| Pastaza | 1995-1999 | 27.15 | 38.7 (30.46-48.45) |
| Pastaza | 2000-2004 | 30.82 | 44.6 (36.27-54.18) |
| Pastaza | 2005-2009 | 43.61 | 60.6 (51.66-70.68) |
| Pastaza | 2010-2014 | 49.07 | 67.4 (58.6-77.1) |
| Pastaza | 2015-2019 | 51.95 | 70.9 (62.51-80.14) |
| Sucumbíos | 1990-1994 | 22.73 | 31.4 (26.08-37.48) |
| Sucumbíos | 1995-1999 | 29.58 | 40.7 (34.87-47.15) |
| Sucumbíos | 2000-2004 | 31.05 | 42.4 (36.83-48.49) |
| Sucumbíos | 2005-2009 | 41.13 | 56.4 (50.32-62.94) |
| Sucumbíos | 2010-2014 | 47.25 | 64.4 (58.32-70.94) |
| Sucumbíos | 2015-2019 | 46.03 | 64.2 (58.38-70.36) |
|  |  |  |  |
