# Supplementary material for: Cancer incidence in the Ecuadorian Amazon: A retrospective study (1990–2019)
Source: PLoS One. 2026 Oct 5;21(10):e0359707. doi: 10.1371/journal.pone.0359707 (PMC13637904; doi:10.1371/journal.pone.0359707)
Supplement: Supplementary file 2 [file pone.0359707.s002.docx]

***APPENDIX 2***

***Table S2. Results of the quasi-Poisson regression analysis of overall cancer incidence by calendar period, age group, and sex, Ecuador, 1990–2019.***

|  | **IRR*** | **95% CI*** |
| --- | --- | --- |
| Period (per 5-year increase) | 1.23 | 1.20-1.25 |
| Women vs Men | 2.00 | 1.87-2.13 |
| Age |  |  |
| 1-4 | 1.46 | 0.89-2.38 |
| 5-9 | 0.69 | 0.42-1.15 |
| 10-14 | 0.87 | 0.53-1.45 |
| 15-19 | 0.95 | 0.57-1.58 |
| 20-24 | 1.76 | 1.08-2.87 |
| 25-29 | 2.57 | 1.59-4.15 |
| 30-34 | 4.82 | 3.01-7.72 |
| 35-39 | 6.44 | 4.03-10.30 |
| 40-44 | 8.57 | 5.36-13.69 |
| 45-49 | 9.79 | 6.13-15.64 |
| 50-54 | 13.73 | 8.60-21.93 |
| 55-59 | 18.14 | 11.36-28.94 |
| 60-64 | 22.96 | 14.38-36.67 |
| 65-69 | 28.22 | 17.67-45.07 |
| 70-74 | 32.69 | 20.39-52.39 |
| 75-79 | 43.80 | 27.17-70.61 |
| 80+ | 38.39 | 23.78-61.98 |

***** IRR: Incidence Rate Ratio; CI: Confidence Interval. Estimates obtained from a quasi-Poisson regression model with the logarithm of the population included as an offset term. The reference categories were men, age <1 year, and the first calendar period. The IRR for period represents the relative change in incidence associated with each successive 5-year period.
